# Erratum: Rufino-Moya, P.J., et al. Methane Production of Fresh Sainfoin, with or without PEG, and Fresh Alfalfa at Different Stages of Maturity is Similar, but the Fermentation End Products Vary. *Animals* 2019, 9, 197

**DOI:** 10.3390/ani9070421

**Published:** 2019-07-05

**Authors:** Pablo José Rufino-Moya, Mireia Blanco, Juan Ramón Bertolín, Margalida Joy

**Affiliations:** Centro de Investigación y Tecnología Agroalimentaria de Aragón (CITA), Instituto Agroalimentario de Aragón–IA2 (CITA-Universidad de Zaragoza), Avda, Montañana 930, 50059 Zaragoza, Spain; pjrufino@cita-aragon.es (P.J.R.-M.); mblanco@aragon.es (M.B.); jrbertolin@cita-aragon.es (J.R.B.)

The authors wish to make the following correction to their paper [1].

In Table 2, the production of methane in alfalfa at the start-flowering should be 38 mL/g dOM and not 3 mL/g dOM.

The authors would like to apologize for any inconvenience caused.

## Figures and Tables

**Table 2:** Effect of the substrate (S) and the stage of maturity ^1^ (SM) on gas and methane production (CH_4_), potential gas production (*A*), rate of gas production (*c*), in vitro organic matter degradability (IVOMD), ammonia (NH_3_-N), and volatile fatty acids (VFAs).

	Alfalfa			Sainfoin			Sainfoin+PEG				P-values		
Item	VEG	Start-F	End-F	VEG	Start-F	End-F	VEG	Start-F	End-F	r.s.d.^2^	S	SM	SxSM
pH	6.42 ^a^	6.44 ^a^	6.41 ^a^	6.35 ^bx^	6.32 ^by^	6.33 ^by^	6.37 ^bx^	6.31 ^bz^	6.34 ^by^	0.032	< 0.001	0.002	0.01
Gas production (mL/g dOM ^3^)	179	184	188	183	163	181	180	199	173	26.5	0.43	0.97	0.12
*A* (mL)	68 ^x^	67 ^bxy^	62 ^y^	63 ^y^	74 ^bx^	70 ^x^	65 ^y^	78 ^ax^	67 ^y^	5.7	0.01	< 0.001	< 0.001
*c* (h^−1^)	0.2 ^a^	0.22 ^a^	0.19	0.14 ^by^	0.16 ^bxy^	0.19 ^y^	0.15 ^bx^	0.15 ^bx^	0.19 ^y^	0.037	< 0.001	0.02	0.05
CH_4_ production (mL/g dOM ^3^)	37	38	39	37	33	38	37	39	36	5.0	0.29	0.73	0.17
CH_4_:gas (%)	14.5	14.2	14.1	14.1 ^xy^	13.4 ^y^	14.8 ^x^	14.5	14.4	14.4	0.88	0.35	0.13	0.11
IVOMD (%)	85.13 ^bx^	83.73 ^bx^	76.97 ^by^	84.97 ^by^	92.59 ^ax^	85.58 ^ay^	86.81 ^ay^	93.59 ^ax^	86.41 ^ay^	1.692	< 0.001	< 0.001	< 0.001
NH_3_-N (mg/L)	240 ^a^	247 ^a^	210	206 ^b^	184 ^b^	201	242 ^a^	234 ^a^	215	31.7	< 0.001	0.06	0.20
Total VFAs (mmol/L)	102 ^xy^	105 ^x^	97 ^y^	96 ^y^	103 ^x^	101 ^xy^	99 ^y^	105 ^x^	102 ^xy^	7.1	0.61	0.02	0.35
Acetic acid (C_2_) (mol/100 mol)	63.1 ^cy^	63.9 ^bx^	64.0 ^bx^	65.4 ^ay^	66.5 ^ax^	66.3 ^ax^	63.9 ^by^	64.6 ^bx^	64.5 ^bx^	0.76	< 0.001	< 0.001	0.95
Propionic acid (C_3_) (mol/100 mol)	15.5 ^a^	15.3 ^a^	15.3 ^a^	15 ^bx^	14.5 ^cy^	14.5 ^by^	15.3 ^ax^	14.9 ^by^	15.2 ^ax^	0.28	< 0.001	< 0.001	0.09
Butyric acid (mol/100 mol)	13.0 ^a^	12.6 ^a^	12.6 ^a^	12.1^c^	12.2 ^b^	12 ^b^	12.5 ^b^	12.8 ^a^	12.4 ^a^	0.45	< 0.001	0.20	0.16
Iso-butyric acid (mol/100 mol)	1.97 ^ax^	1.91 ^axy^	1.87 ^ay^	1.82 ^bx^	1.68 ^by^	1.72 ^by^	1.97 ^ax^	1.83 ^ay^	1.84 ^ay^	0.100	< 0.001	< 0.001	0.63
Valeric acid (mol/100 mol)	2.14 ^axy^	2.09 ^ay^	2.22 ^ax^	1.87 ^bx^	1.7 ^cy^	1.83 ^cx^	2.12 ^ay^	1.95 ^bx^	2.04 ^by^	0.107	< 0.001	< 0.001	0.11
Iso-valeric acid (mol/100 mol)	4.27 ^ax^	4.09 ^axy^	4.04 ^ay^	3.87 ^bx^	3.5 ^by^	3.65 ^by^	4.19 ^ax^	3.91 ^ay^	3.93 ^ay^	0.223	< 0.001	< 0.001	0.69
C_2_:C_3_ (mol/mol)	4.1 ^cy^	4.18 ^cxy^	4.2 ^bx^	4.39 ^ay^	4.62 ^ax^	4.59 ^ax^	4.18 ^by^	4.36 ^bx^	4.25 ^by^	0.109	< 0.001	< 0.001	0.13
CH_4_:VFA (mL/mol)	2.41	2.24 ^b^	2.35	2.53	2.22 ^b^	2.48	2.62	2.73 ^a^	2.4	0.313	0.01	0.26	0.10

^1^ VEG: vegetative; Start-F: start of flowering; End-F: end of flowering; ^2^ residual standard deviation; ^3^ degraded organic matter. Within a parameter, means with different superscript (a,b,c) differ at *P <* 0.05 for the substrate effect in each stage of maturity; with different superscript (x,y,z) at *P <* 0.05 for the stage of maturity effect in each substrate.
